# TAp63γ and ΔNp63β Promote Osteoblastic Differentiation of Human Mesenchymal Stem Cells: Regulation by Vitamin D3 Metabolites

**DOI:** 10.1371/journal.pone.0123642

**Published:** 2015-04-07

**Authors:** Kevin M. Curtis, Kristina K. Aenlle, Rachel N. Frisch, Guy A. Howard

**Affiliations:** 1 Geriatric Research, Education, and Clinical Center and Research Service, Bruce W. Carter Veterans Affairs Medical Center, Miami, Florida, United States of America; 2 Department of Biochemistry and Molecular Biology, University of Miami Miller School of Medicine, Miami, Florida, United States of America; 3 Department of Medicine, University of Miami Miller School of Medicine, Miami, Florida, United States of America; University of Massachusetts Medical, UNITED STATES

## Abstract

The transcription factor p63 is required for skeletal formation, and is important for the regulation of 1α,25(OH)_2_D_3_ receptor (VDR) in human mesenchymal stem cells (hMSC). Herein we report that TAp63γ and ΔNp63β appear to be an integral part of the osteoblastic differentiation of hMSC and are differentially regulated by the vitamin D_3_ metabolites 1α,25(OH)_2_D_3_ and 24R,25(OH)_2_D_3_. We compared the endogenous expression of p63 isoforms (TA- and ΔNp63) and splice variants (p63α, -β, -γ), in naive hMSC and during osteoblastic differentiation of hMSC. TAp63α and -β were the predominant p63 variants in naive, proliferating hMSC. In contrast, under osteoblastic differentiation conditions, expression of p63 changed from the TAp63α and -β to the TAp63γ and ΔNp63β variants. Transient overexpression of the p63 variants demonstrated that TAp63β, ΔNp63β, and ΔNp63γ increased alkaline phosphatase activity and ΔNp63α and -γ increased the expression of mRNA for *osteocalcin* and *osterix*. Our results support the hypothesis that TAp63α and -β promote a naive state in hMSC. Moreover, TAp63γ is increased during and promotes early osteoblastic differentiation through the expression of pro-osteogenic genes; *VDR*, *Osterix*, *Runx2* and *Osteopontin*. ΔNp63β also appears to support osteogenic maturation through increased alkaline phosphatase activity. Treatment with 1α,25(OH)_2_D_3_ increased the expression of mRNA for ΔNp63, while addition of 24R,25(OH)_2_D_3_ increased the expression of TA- and ΔNp63γ variants. These novel findings demonstrate for the first time that p63 variants are differentially expressed in naive hMSC (TAp63α,β), are important during the osteoblastic differentiation of hMSC (TAp63γ and ΔNp63β), and are differentially regulated by the vitamin D3 metabolites, 1α,25(OH)_2_D_3_ and 24R,25(OH)_2_D_3_. The molecular nuances and mechanisms of osteoblastic differentiation presented here will hopefully improve our understanding of bone development, complications in bone repair (mal- and non-union fractures), osteoporosis and possibly lead to new modalities of treatment.

## Introduction

The transcription factor p63, a member of the p53 family of transcription factors, is required for proper skeletal formation in development [1]. While p63 is structurally similar to p53, the effects of p63 on growth and differentiation have been demonstrated to be distinct from the rest of the p53 family of transcription factors (see review; [2]). The expression of the gene for p63 is complex, involving differential promoter selection and carboxyl-terminal alternative RNA splicing to generate various p63 isoforms with different activities [2]. Two sets of p63 isoforms are produced: TAp63 isoforms (containing a transactivating domain and a complete NH2 terminus) and truncated ΔNp63 isoforms (lacking NH2 terminus). In addition, mRNA splicing results in different C-terminal lengths producing α, β, and γ variants, thus generating 6 different variants: TAp63α,β,γ and Δp63α,β,γ. The TA versus ΔN isoforms of p63 can act in opposition to each other, either to activate or repress growth and differentiation [2]. For example, during development the expression of TAp63 is required for epithelial stem cell maintenance, while increased ΔNp63 expression promotes terminal differentiation [3]. This suggests a novel role for TA/ΔNp63 isoform modulation in stem cell self-renewal and differentiation. Therefore, this is the first study examining the role of the p63 variants during osteoblastic differentiation of primary human mesenchymal stem cells (hMSC).

Mesenchymal stem cells are a naive population of adult stem cells residing in a specialized bone niche, giving rise to osteoblasts, chondrocytes, and adipocytes during development and throughout mammalian adult life. Naive hMSC are known to differentiate into matrix-producing, bone-forming osteoblasts. Bone formation not only requires osteoblasts, but is a concerted process involving chondrocytes and hypertrophic chondrocytes as well. The biological significance of the TA/ΔNp63 (α, β, γ) variants during the osteoblastic differentiation of hMSC and their importance for hMSC-mediated bone repair are not established. The regulatory relationship between p63 and the actions of 1α,25(OH)_2_D_3_ / 24R,25(OH)_2_D_3_ during osteoblastic differentiation of hMSC are also not fully understood and have yet to be studied.

Initial studies in total p63 knock-out mice demonstrated decreased endochondral bone formation, suggesting that p63 is required for this process [1,4]. Additionally, follow-up studies with overexpression of TAp63α (*Col10a1-*TApcα) in the hypertrophic chondrocytes of mice showed accelerated ossification during late embryonic skeletal development and an overall increase in osteoblast mediated mineralization [5]. This suggested a function for TAp63α during endochondral bone formation, possibly due to increased chondrocyte maturation or apoptosis [5]. In contrast, the overexpression of ΔNp63α (C*ol10a1*-ΔNp63α) in hypertrophic chondrocytes of mice did not lead to abnormalities during embryonic skeletal development [6]. In addition, overexpression of both TAp63α and ΔNp63α using the chondrocyte specific *Col2a1* promoter, demonstrated that TAp63α is involved in endochondral bone formation and ossification, while ΔNp63α is not [6]. Furthermore, in mouse hypertrophic chondrocyte cell lines (MCT and ATDC5) undergoing differentiation *in vitro*, there was mRNA expression of the TA63γ variant in proliferating cells and was found to increase during differentiation [7]. Taken together, these findings [1,4–6] suggest that even though TAp63α overexpression results in increased skeletal formation in mice, other p63 variants such as TAp63γ, may also be involved [7]. These previous studies demonstrate that p63 is involved in endochondral bone formation through the regulation of chondrogenesis, impacting endochondral ossification and bone formation; however, the role of p63 during osteoblastic differentiation of hMSC is still unclear.

Bone homeostasis and repair are regulated by several vitamin D_3_ metabolites. Of these 1,25-dihydroxyvitamin D_3_ (1α,25(OH)_2_D_3_) and 24R,25-dihydroxyvitamin D_3_ (24R,25(OH)_2_D_3_) are considered the most important [8]. However, the mechanism(s) by which 1α,25(OH)_2_D_3_ and 24R,25(OH)_2_D_3_ differentially mediate the osteoblastic differentiation of hMSC is not thoroughly understood. Recent work has demonstrated that p63 is involved in the expression of the 1α,25(OH)_2_D_3_- vitamin D_3_ receptor (VDR) in hMSC, which is important for the osteoblastic differentiation, by the cooperative action of hepatocyte growth factor and 1α,25(OH)_2_D_3_ [9]. This further raises questions about the regulatory role of p63 during the osteoblastic differentiation of hMSC.

The initial aim of this study assessed the basal expression pattern of p63 gene products in naive hMSC, compared to hMSC undergoing osteoblastic differentiation. This was done through the analysis of (1) p63 gene expression using RT-qPCR to distinguish between the mRNA expression of the *TAp63* versus *ΔNp63* isoforms and the splice variants (*α* and *β* versus *γ*) and (2) western blot analysis to determine changes in protein expression. The second aim focused on the differential mRNA and protein expression of the p63 gene products after treatment with the vitamin D_3_ metabolites 1α,25(OH)_2_D_3_ and 24R,25(OH)_2_D_3_. The third aim focused on determining which *p63* gene product(s) is/are important for the osteoblastic differentiation of hMSC by (1) transient and (2) stable overexpression of all six *p63* gene products (TAp63α, -β, -γ and ΔNp63α, -β, -γ) and (3) the knockdown of total p63 or targeted knockdown of specific p63 variants. After overexpression or knockdown of p63 gene products, and induction of osteoblastic differentiation, alkaline phosphatase activity and RT-qPCR analysis of mRNA expression of pro-osteogenic genes (*VDR*, *osteocalcin*, *osterix*, *osteopontin* and *runx2*) was determined. As a final aim, and to further challenge the idea that p63 undergoes gene changes during the differentiation process, we transitioned hMSC from 21% to 3% O_2_ and then assessed mRNA and protein expression of p63. Low oxygen tension (1–3% O_2_) has been shown to inhibit hMSC differentiation while maintaining hMSC in a naive state, while higher oxygen (normoxia, 21% O_2_), as used in these studies, promotes differentiation [10,11].

These studies demonstrate for the first time that p63 gene products are important for the osteoblastic differentiation of hMSC, and help elucidate the mechanism through which the vitamin D_3_ metabolites exert their pro-osteogenic effects.

## Results

### TAp63α and -β are the predominant endogenous p63 gene products in naive hMSC

**Fig 1:**
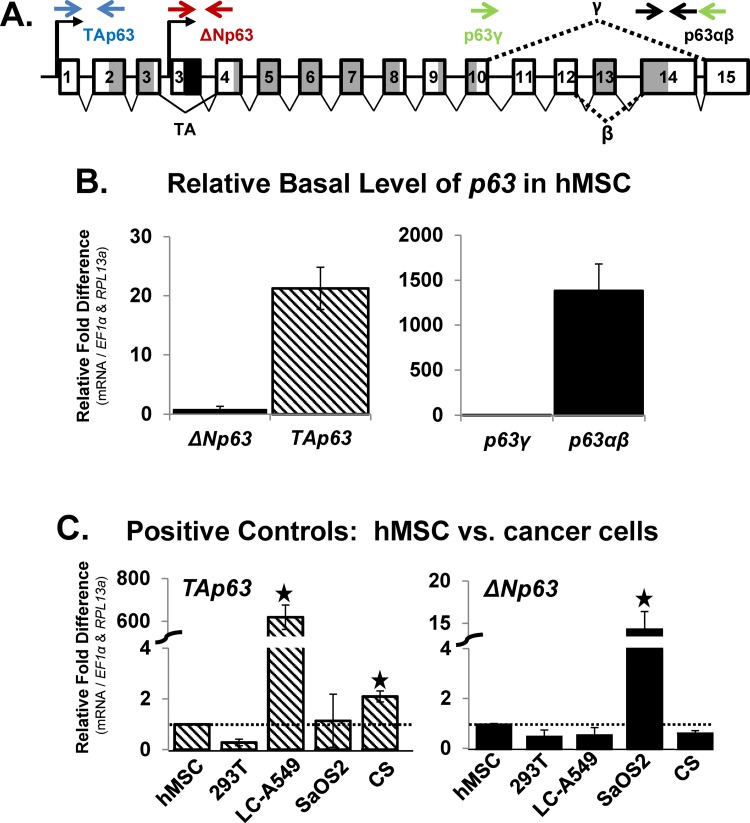
TAp63α and -β are the predominant endogenous p63 gene products in naive hMSC. A) Schematic diagram (modified from [38]) depicting the p63 gene structure with the exons numbered sequentially and the relative location of primer pairs versus p63 exons used for RT-qPCR analysis of mRNA expression of the p63 mRNA isoforms (*TA-* and *ΔNp63*) and splice variants (*p63α* / *β* and p63*γ*). B) hMSC were seeded at low-density (1,000 cells/cm^2^) and grown under expansion conditions to maintain the pool of naive hMSC. RT-qPCR analysis of mRNA expression of the p63 isoforms (left panel; *TA-* and *ΔNp63*) and splice variants (right panel; *p63α* / *β* and *p63γ*) under expansion conditions, expressed as relative fold difference. The real-time calculated PCR primer pair efficiency was calculated for each primer pair set in order to compare the relative fold difference in p63 variant expression in hMSC. C) RT-qPCR analysis of mRNA expression comparing hMSC versus various cancer cell lines was used to validate p63 primer pair sets and compare p63 expression in hMSC versus known expression in cancer cells as a positive control. Cell lines: human embryonic kidney (293T), non-small cell lung cancer (LC-A549), osteosarcoma (SaOS2); chondrosarcoma primary cell culture (CS). N = 3 independent experiments in triplicate. (*) p ≤0.05 compared to (B) left panel: *ΔNp63*, right panel: *TAp63* or (C) hMSC, which were set to the value of “1”. hMSC used were from a 7 and 22 year old male.

Initial studies were performed to determine the mRNA expression profile of the endogenous p63 variants in naive hMSC grown under non-confluent (1,000 cells/cm^2^) expansion conditions. Primer pair sets were created that distinguished between the alternative exons for TAp63 versus ΔNp63 variants, or for the unique carboxyl-terminus of the three p63 splice variants (α,β,γ). These primer pair sets detected TAp63α,β,γ **(TAp63)** versus ΔNp63α,β,γ **(ΔNp63)** or detected TA/ΔNp63α,β **(p63α,β)** versus TA/ΔNp63γ **(p63γ)** (Fig 1A; see schematic diagram, arrows represent primer pair sets). In naive hMSC, mRNA expression of *TAp63* was higher than *ΔNp63* (Fig 1B; left). mRNA expression of *p63α*,*β* was higher than *p63γ* (Fig 1B; right), suggesting that *TAp63α*,*β* are the predominant mRNA variants found in naive hMSC.

As positive controls, RT-qPCR analysis was used to compare mRNA expression of p63 variants in hMSC versus that in human embryonic kidney (293T), non-small cell lung cancer (LC-A549), osteosarcoma (SaOS2) cell lines, and chondrosarcoma (CS) primary cell cultures. The mRNA expression of *TAp63* was significantly (p<0.05; >600 fold) higher in LC-A549 compared to hMSC. The mRNA expression of *TAp63* in hMSC was similar to 293T, SaOS2 and CS (Fig 1C; left). The mRNA expression of *ΔNp63* was significantly (p<0.05) higher in SaOS2 compared to hMSC, while the mRNA expression of *ΔNp63* in hMSC was similar to 293T, LC-A549 and CS cells (Fig 1C; right). Due to their high levels of expression, LC-A549 and SaOS2 cells were subsequently used as positive controls for RNA and protein analysis of TAp63 and ΔNp63, respectively, throughout the remainder of the studies presented.

### The mRNA expression of p63 variants is dependent on seeding cell density of hMSC

The *in vitro e*xpansion of hMSC was done at low cell densities, ranging from 500–1,000 cells/cm^2^, allowing for the maintenance of a naive undifferentiated phenotype [12,13]. During *in vitro* osteoblastic differentiation of hMSC, higher cell densities were used (1,000–30,000 cells/cm^2^ [8,12,14]). The mRNA expression levels of the p63 variants were assessed to determine if there was a difference in expression between expansion (low cell densities) and osteoblastic differentiation conditions (high cell densities).

**Fig 2:**
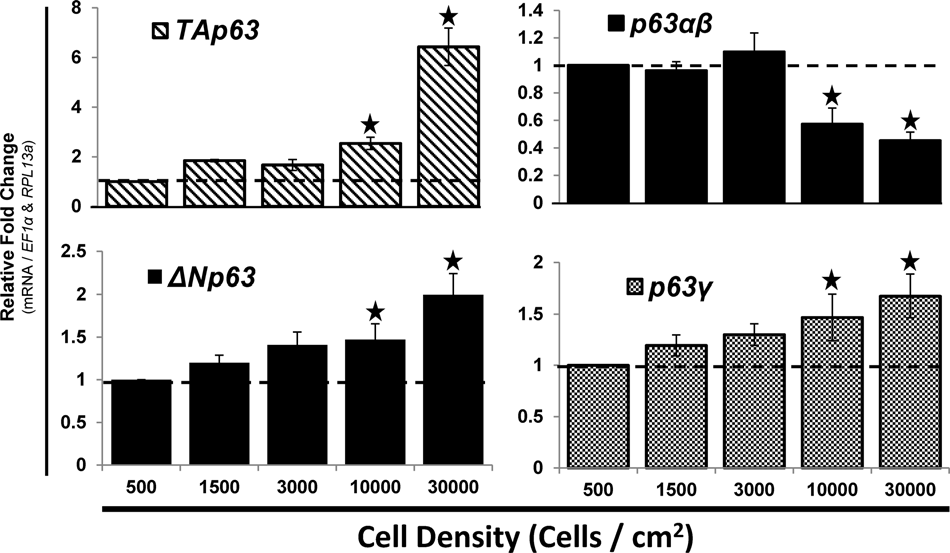
The mRNA expression of p63 gene variants is dependent on seeding cell density of hMSC. hMSC were seeded at various densities, ranging from 500–30,000 cell/cm^2^, and then grown under expansion conditions for 3-days followed by RT-qPCR analysis of p63 gene products. RT-qPCR analysis of p63 isoforms (left panel, *TA-* and *ΔNp63*) and splice variant mRNA (right panel, *p63α* / *β* and p63*γ*) expression. N = 3 independent experiments in triplicate. (*) p ≤ 0.05 compared to hMSC seeded at 500 cells/cm^2^, which were set to the value of “1”. hMSC used were from a 7 and 22 year old male.

After a 3-day expansion/culture period, the cells were harvested to determine if there were changes in the mRNA expression of p63. At 10,000–30,000 cells/cm^2^ the mRNA expression levels of *TAp63* were significantly greater (>2-fold at 10,000 cells/cm^2^ and 6-fold at 30,000 cells/cm^2^) relative to the cells seeded at 500 cell/cm^2^ (Fig 2; top left). The mRNA expression levels of *ΔNp63* were significantly (p<0.05) higher (approximately 2-fold) in cells seeded at 10,000 or 30,000 cells/cm^2^ compared to cells seeded at 500 cells/cm^2^ (Fig 2; bottom left). The levels of *p63αβ* were significantly lower (p<0.05) (Fig 2; top right), while the levels of *p63γ* were significantly higher (p<0.05) (Fig 2; bottom right) in cells seeded at 10,000 or 30,000 cells/cm^2^ compared to the levels in cells seeded at 500 cells/cm^2^. These differences suggest that mRNA alternative splicing of *p63* changes when cells are plated at effectively confluent densities. In order to maintain a stable p63 expression profile, cell confluence, and minimize cell proliferation during osteoblastic differentiation of hMSC, 10,000 cells/cm^2^ was used for all osteoblastic differentiation studies presented herein.

### Vitamin D_3_ metabolite 1α,25(OH)_2_D_3_ increased the mRNA expression of *Δ* *Np63* while 24R,25(OH)_2_D_3_ altered the mRNA splice variant expression of *p63γ* during expansion

**Fig 3:**
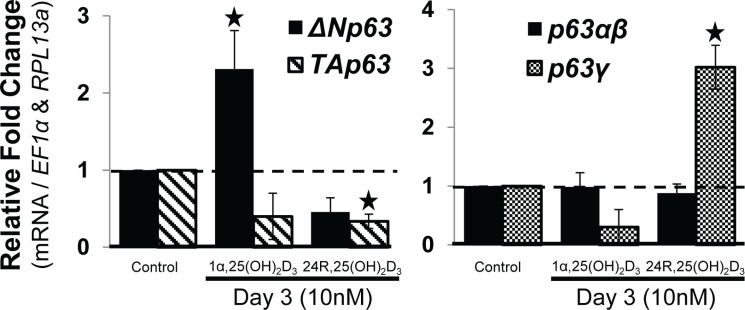
Vitamin D_3_ metabolite 1α,25(OH)_2_D_3_ increased mRNA expression of ***Δ***Np63, while 24R,25(OH)_2_D_3_ altered mRNA splice variant expression of p63γ during expansion. hMSC were seeded at 1,000 cells/cm^2^ and grown under expansion conditions. hMSC were then treated (or not = control) once with the vitamin D_3_ metabolites (Day 0) 1α,25(OH)_2_D_3_ (10nM) or 24R,25(OH)_2_D_3_ (10nM) for a 3-day period prior to RT-qPCR analysis of p63 gene products. RT-qPCR analysis of p63 isoforms (left panel, *TA-* and *ΔNp63*) and splice variant mRNA (right panel, *p63α* / *β* and p63*γ*) expression. N = 3 independent experiments in triplicate. (*) p ≤0.05 compared to control (expanded, untreated hMSC). hMSC used were from a 7 and 22 year old male.

The active vitamin D_3_ metabolites 1α,25(OH)_2_D_3_ and 24R,25(OH)_2_D_3_ have both been shown to play a role during osteoblastic differentiation [8], yet their mechanisms are not fully understood. Treatment with 1α,25(OH)_2_D_3_ during the expansion of hMSC led to a significant (p<0.05) increase in mRNA expression of *ΔNp63*, whereas treatment with 24R,25(OH)_2_D_3_ did not increase *ΔNp63* (Fig 3; left). 1α,25(OH)_2_D_3_ appeared to decrease the mRNA expression of *TAp63*, although this did not reach significance (p<0.06). Neither 1α,25(OH)_2_D_3_ nor 24R,25(OH)_2_D_3_ increased the mRNA expression of *TAp63*. However, vitamin D_3_ metabolites did effect the mRNA alternative splicing of p63: 24R,25(OH)_2_D_3_ significantly (p<0.05) increased mRNA expression of *p63γ*, while no effect was observed on *p63α*,*β* variants. Conversely, 1α,25(OH)_2_D_3_ appeared to decrease the mRNA expression of *p63γ*, although not significantly. (Fig 3; right).

### TAp63γ and ΔNp63β are the predominant p63 variants during osteoblastic differentiation of hMSC

**Fig 4:**
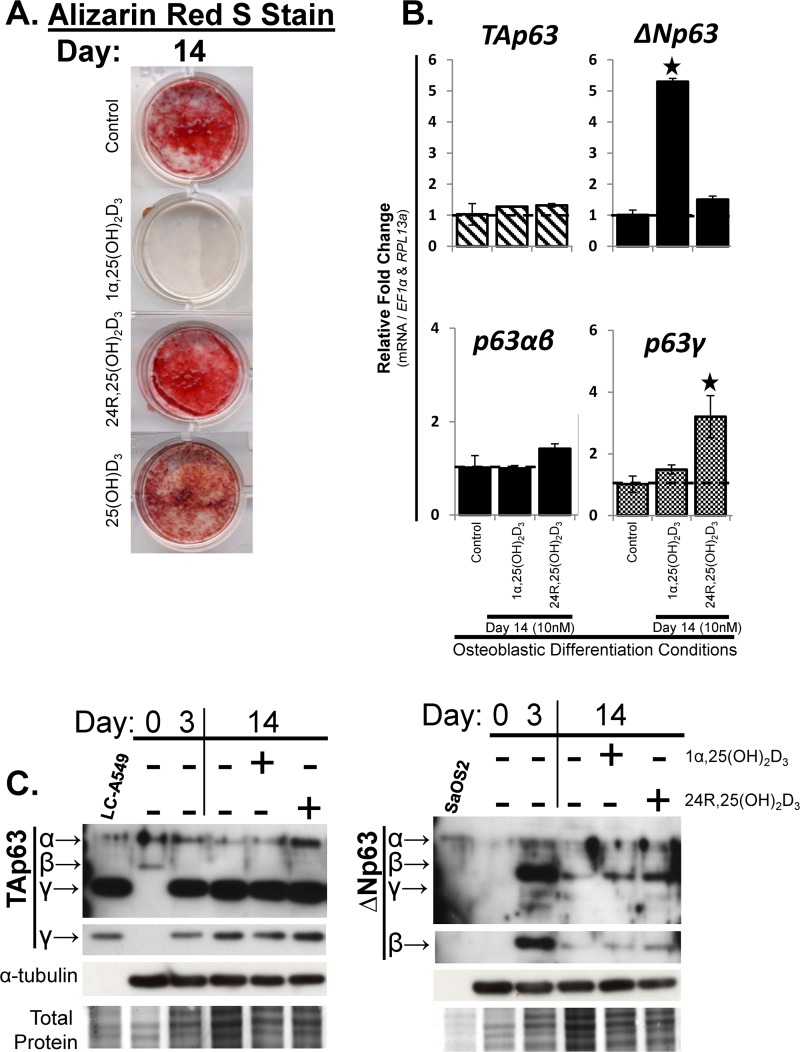
TAp63γ and ΔNp63β are the predominant p63 variants during osteoblastic differentiation of hMSC. hMSC were seeded at 10,000 cells/cm^2^ under expansion conditions overnight then switched to osteoblastic differentiation conditions, without dexamethasone (Day 0). hMSC were treated with vitamin D_3_ metabolites 1α,25(OH)_2_D_3_ or 24R,25(OH)_2_D_3_ (10nM), or the vitamin D_3_ pro-hormone 25-hydroxyvitamin D_3_ (25(OH)D_3_) (10nM), starting at Day 0, and then re-treated every 3 days with media changes through Day 14. Control groups had no vitamin D_3_ treatments. Cells were fixed or harvested for RNA and protein at Days 3 and 14. A) Alizarin Red-S stain was used to determine Ca^2+^ mineralization. B) RT-qPCR analysis of p63 isoform (top panels, *TA-* and *ΔNp63*) and splice variant mRNA (bottom panels, *p63α* / *β* and p63*γ*) expression. C) Western blot analysis of p63 isoforms using antibodies detecting TAp63 variants (left panel, TAp63α, β, γ) or ΔNp63 variants (right panel, ΔNp63α, β, γ) during expansion conditions (Day 0) and under osteoblastic differentiation conditions (Days 3 and 14). Coomassie Blue = Total protein. Positive controls: TAp63: LC-A549 cells (positive for TAp63γ and low-expression of TAp63α protein) and ΔNp63; SaOS2 cells (positive for ΔNp63 variants). N = 3 independent experiments in triplicate. (*) p ≤0.05 compared to control (expanded, untreated hMSC). hMSC used were from a 7 and 22 year old male.

1α,25(OH)_2_D_3_ and 24R,25(OH)_2_D_3_ have unique roles during osteoblastic differentiation and Ca^2+^ mineralization, which is a hallmark of matrix producing osteoblasts [8]. During *in vitro* osteoblastic differentiation, hMSC undergo Ca^2+^ mineralization (Fig 4A; control). During *in vitro* osteoblastic differentiation, treatment of hMSC with 1α,25(OH)_2_D_3_ inhibited, while 24R,25(OH)_2_D_3_ induced Ca^2+^ mineralization (as previously described [8]) (Fig 4A). For the course of our studies we did not use dexamethasone for *in vitro* osteoblastic differentiation, as it is not required for Ca^2+^ mineralization *in vitro* [8], and *in vivo* has been shown to induce glucocorticoid-induced bone loss [15].

During osteoblastic differentiation of hMSC, 1α,25(OH)_2_D_3_ significantly (p<0.05) increased mRNA expression of *ΔNp63*, while 24R,25(OH)_2_D_3_ had no effect (Fig 4B). Treatment with 1α,25(OH)_2_D_3_ or 24R,25(OH)_2_D_3_ did not increase mRNA expression of *TAp63* (Fig 4B). Neither 1α,25(OH)_2_D_3_ nor 24R,25(OH)_2_D_3_ had any effect on mRNA expression of *p63α*,*β*, whereas 24R,25(OH)_2_D_3_ treatment significantly (p<0.05) increased *p63γ* (Fig 4B). Assessment of p63 protein levels revealed that naive hMSC (Day 0 of *in vitro* osteoblastic differentiation) expressed predominantly TAp63α,β protein (Fig 4C; left) and mRNA (Fig 1B), while there was no detectable protein expression of ΔNp63 (Fig 4C; right). After 3-days of osteoblastic differentiation conditions, there was a switch in protein expression of p63 from TAp63α,β to predominantly TAp63γ (Fig 4C; left), which was maintained throughout the 14-day osteoblastic differentiation.

Because TAp63γ was undetectable at Day 0, the Day 3 value was set to a value of “1” to facilitate evaluation of the increase observed at Day 14 (1.54 relative increase). Note that there was no subsequent difference in TAp63γ expression as a results of 1α,25(OH)_2_D_3_ or 24R,25(OH)_2_D_3_ treatment on Day 14 (Fig 4C; left). Protein expression of ΔNp63 increased dramatically from Day 0 to Day 3, and then appeared to decrease slightly between Day 3 (set to the value of “1”) and Day 14 (0.09 relative decrease). Treatment with 1α,25(OH)_2_D_3_ did not alter the expression of ΔNp63β, while treatment with 24R,25(OH)_2_D_3_ appeared to maintain or slightly increase ΔNp63β protein expression (3.81 relative increase) as compared to Day 14 (Fig 4C; right). Treatment with 24R,25(OH)_2_D_3_ significantly increased the protein expression of TAp63α by day 14 (7.81 relative increase), as compared to only a 1.62 relative increase by 1α,25(OH)_2_D_3_ (values relative to un-treated Day 14 value set to the value of “1”) (Fig 4C; right).

### Transient overexpression of ΔNp63β, -γ and TAp63β increase alkaline phosphatase

**Fig 5:**
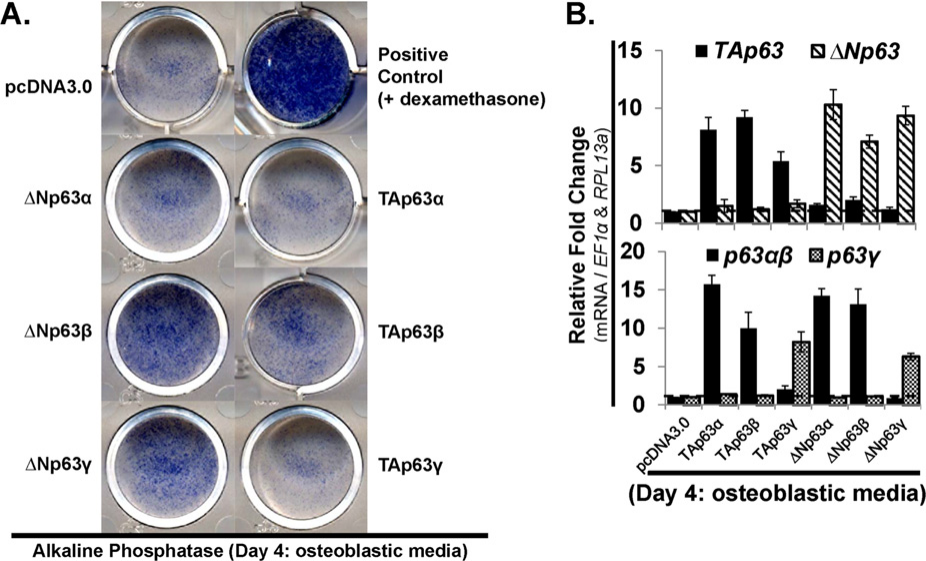
Transient overexpression of ΔNp63β, -γ and TAp63β increase alkaline phosphatase. hMSC were transfected with pcDNA3.0-p63 vectors (7 days), leading to transient overexpression of each of the six p63 variants. hMSC were then switched to osteoblastic differentiation media, no dexamethasone (4 days—post 7-day transfection). A) Alkaline phosphatase stain (Day 4 in osteoblastic differentiation media). Positive control for alkaline phosphatase consisted of osteoblastic differentiation media supplemented with dexamethasone (10nM). B) RT-qPCR analysis of p63 isoform (top panel, *TA-* and *ΔNp63*) and splice variant mRNA (bottom panel, *p63α* / *β* and p63*γ*) expression. RT-qPCR analysis demonstrated the relative level of overexpression of each of the six p63 variants. A & B) Changes in alkaline phosphatase and mRNA expression of p63 variants are compared to hMSC transfected with the empty vector (pcDNA3.0). N = 3 independent experiments. RT-qPCR analysis was also used to assess the mRNA expression of the osteoblastic markers *runx2*, *osterix*, *osteocalcin*, *osteopontin*, *VDR* and the late osteoblastic marker, *BSP*. No change above 2-fold was observed (data not shown). hMSC used were from a 22 year old male.

Following up on our previous findings, which suggested that p63 plays a role during osteoblastic differentiation of hMSC, we transiently overexpressed all six p63 gene products (vector; pcDNA3.0) under expansion conditions. hMSC overexpressing p63 were seeded at 10,000 cell/cm^2^ for osteoblastic differentiation, as described above (Fig 2). Overexpression of TAp63β led to an increase in alkaline phosphatase staining as compared to control (Fig 5A). Moreover, overexpression of both ΔNp63β and ΔNp63γ also led to a more robust increase in alkaline phosphatase staining (Fig 5A). Dexamethasone was used as a positive control due to its known stimulation of alkaline phosphatase (Fig 5A; top right). RT-qPCR analysis was used to assess the mRNA expression of each of the six groups transiently overexpressing p63 gene products (Fig 5B). We were able to observe ~6–15 fold increases in expression of all six p63 variants. RT-qPCR analysis was also used to assess mRNA expression of the osteoblastic markers *runx2*, *osterix*, *osteocalcin*, *osteopontin*, *VDR* and the late osteoblastic marker, *BSP*. Although we were able to obtain measurable values for each of these markers, no change above 2-fold was observed for any of them (data not shown). Also of note, overexpression of TAp63β, TAp63γ and ΔNp63β completely blocked cell proliferation (data not shown).

### Stable overexpression of ΔNp63α and ΔNp63γ increased the mRNA expression of osteocalcin and osterix, while decreasing VDR

**Fig 6:**
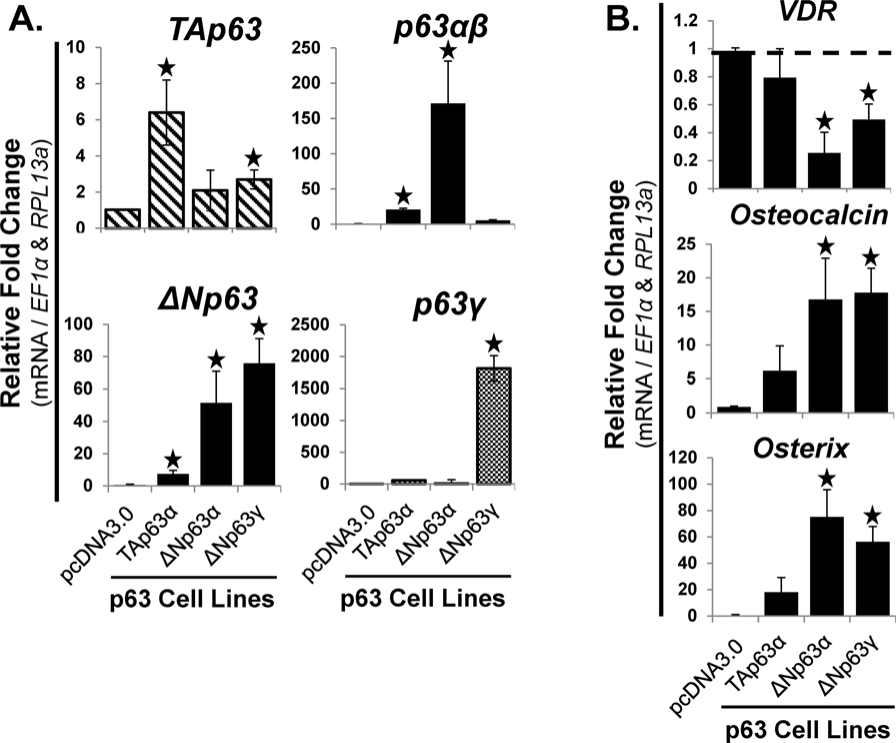
Stable overexpression of **Δ**Np63α and **Δ**Np63γ increased the mRNA expression of osteocalcin and osterix, and decreased VDR. hMSC were transfected with pcDNA3.0-p63 vectors (7 days), followed by selection with G418 (3 weeks). The cells were then expanded and cloned over 2 months. Overexpression of TAp63β, TAp63γ and ΔNp63β caused a cell proliferation arrest; hence they were not available for long-term expansion and analysis. A) RT-qPCR analysis of p63 isoform (left panels, *TA-* and *ΔNp63*) and splice variant mRNA (right panels, *p63α* / *β* and p63*γ*) expression. RT-qPCR analysis demonstrated the relative level of overexpression of each of the p63 variants compared to empty vector (pcDNA3.0). B) RT-qPCR analysis of mRNA expression of osteoblastic differentiation markers: *VDR* (vitamin D_3_ receptor (binds 1α,25-dihydroxyvitamin D_3_)), *Osteocalcin*, and *Osterix*. Also of note, the overexpression of TAp63α, ΔNp63α, and ΔNp63γ did not alter the mRNA expression of *runx2*, *osteopontin* or the late stage osteoblastic gene, *BSP*. N = 3 independent experiments in triplicate. (*) p ≤ 0.05 compared to control (hMSC with empty vector, pcDNA3.0). hMSC used were from a 22 year old male.

To further assess the effects of p63 on osteoblastic differentiation, stable primary hMSC cultures overexpressing TAp63α, ΔNp63α or ΔNp63γ were created. Unfortunately, TAp63β, TAp63γ and ΔNp63β completely blocked cell proliferation, hence selection was not possible and we could not create stable primary hMSC cultures of those variants (data not shown). RT-qPCR analysis was used to validate the overexpression of the p63 gene products in each of the stable primary hMSC cultures (Fig 6A). Stable overexpression of ΔNp63α and ΔNp63γ significantly (p<0.05) increased the mRNA expression of *osteocalcin* and *osterix*, and significantly (p<0.05) decreased *VDR* (1α,25(OH)_2_D_3_ receptor) (Fig 6B). Overexpression of TAp63α did not significantly change the mRNA expression of *VDR*, *osteocalcin*, or *osterix* (Fig 6B). Also of note, the overexpression of TAp63α, ΔNp63α, or ΔNp63γ did not alter the mRNA expression of *runx2* or *osteopontin*. Additionally, the mRNA expression of the late stage osteoblastic gene, bone sialoprotein (*BSP*) did not change (data not shown).

### Targeted knockdown of p63γ decreased the mRNA expression of VDR, osterix, runx2 and osteopontin

**Fig 7:**
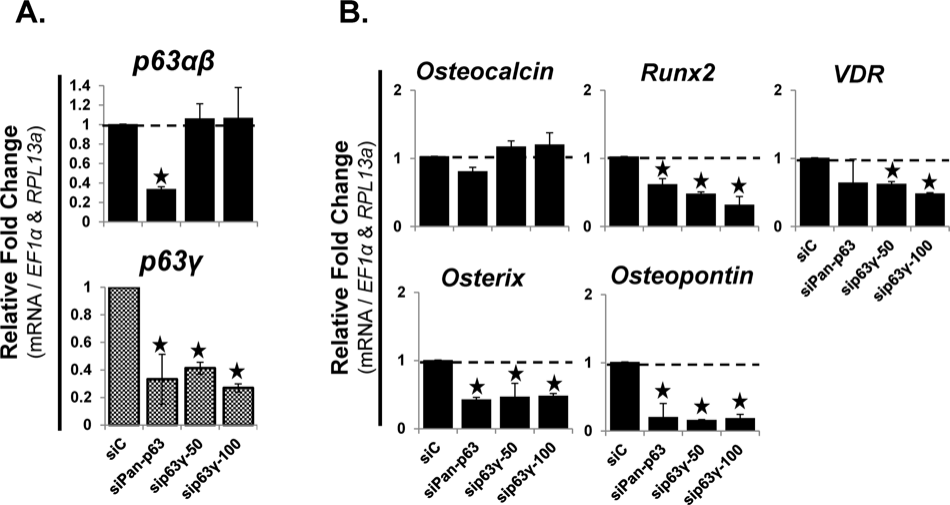
Targeted knockdown of p63γ variants decreased the mRNA expression of VDR, osterix, runx2 and osteopontin. Knockdown of total p63 (siPan-p63; 50nM) or the p63γ (TAp63γ & ΔNp63γ) splice variants (sip63γ; 50nM or 100nM) in hMSC was done using transfection with siRNA (3 days). Osteogenic differentiation media was added on day-3 for 4 additional days; therefore, cells were collected 7 days post siRNA transfection. A) RT-qPCR analysis of mRNA expression of p63 splice variants (top panel, *p63α* / *β*, bottom panel *p63γ*). RT-qPCR analysis of mRNA expression demonstrated the relative level of knockdown of the p63 splice variants by siPan-p63 & sip63γ compared to a scrambled control (siC). B) RT-qPCR analysis of mRNA expression of osteoblastic differentiation markers: *VDR* (vitamin D_3_ receptor (binds 1α,25-dihydroxyvitamin D_3_)), *Osteocalcin*, *Osterix*, *Osteopontin*, *Runx2* and *BSP*. No change was observed for *BSP* (data not shown). Alkaline phosphatase staining was also performed but no change was observed (data not shown). N = 3 independent experiments in duplicate. (*) p ≤ 0.05 compared to control (siC) which were set to the value of “1”. hMSC used were from a 22 year old male.

During the osteoblastic differentiation of hMSC, TAp63γ is the predominant p63 variant expressed (Fig 4C). To validate the role of TAp63γ during the early stages of osteoblastic differentiation of hMSC, siRNA was used to knockdown all p63 variants (siPan-p63) or targeted against the unique carboxyl-terminus of p63γ, knocking down both TAp63γ and ΔNp63γ (sip63γ). RT-qPCR analysis was used to validate the knockdown of the p63 gene products (Fig 7A). The mRNA expression of both *TA-* and *ΔNp63α*,*β* and *p63γ* variants were knocked down by siPan-p63 (50nM), while only *p63γ* was knocked down by sip63γ (50 & 100nM) (Fig 7A). The mRNA expression of early osteoblastic genes, *runx2*, *osterix* and *osteopontin* were all significantly (p<0.05) decreased after knockdown of both total p63 and p63γ (Fig 7B). The mRNA expression of *VDR* (1α,25(OH)_2_D_3_ receptor) was significantly (p<0.05) decreased after knockdown of p63γ, but not after total p63 knockdown (Fig 7B). The late stage osteoblastic gene, *osteocalcin*, was not significantly reduced after total p63 or p63γ knockdown (Fig 7B). Additionally, mRNA expression of the late stage osteoblastic gene, bone sialoprotein (*BSP*) did not change, nor was there any change observed in alkaline phosphatase staining (data not shown).

### Low oxygen decreased the mRNA expression of *TAp63* and *p63γ*

**Fig 8:**
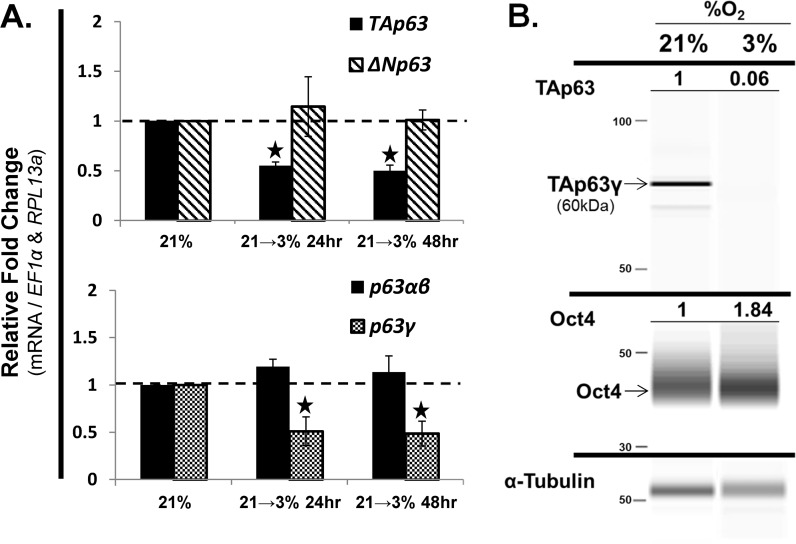
Low oxygen decreased TAp63 and p63γ mRNA expression. hMSC were seeded at 1,000 cells/cm^2^ and grown under expansion conditions in a normoxic atmosphere (21% O_2_), and then switched to low oxygen (3% O_2_) for 24 or 48 hours (A) or grown in 3% O_2_ for 1 week and then passaged once (B). A) RT-qPCR analysis of mRNA expression of p63 isoforms (top panel, *TA-* and *ΔNp63*) and splice variants (bottom panel, *p63α* / *β* and *p63γ*). B) Assessment of protein levels of TAp63 using the Simon SimpleWestern System (ProteinSimple, see Methods). Oct4 is known to increase under low oxygen conditions and was used as a positive control. α-tubulin was used as an internal loading control to quantify the percent change in the protein expression levels of TAp63 and Oct4 (percent change, normalized to α-tubulin, denoted above each lane). Molecular weight of TAp63 corresponds with TAp63γ (60kDa). Molecular weight of Oct4 corresponds to the Oct4 splice varaint Oct4a (~40kDa). N = 3 independent experiments in triplicate. (*) p ≤ 0.05 compared to hMSC expanded under normoxic conditions (21%). hMSC used were from a 7 and 22 year old male.

Low oxygen tension (1–3% O_2_) has been shown to inhibit hMSC differentiation and maintain hMSC in a naive state, while higher oxygen (normoxia, 21% O_2_), as used in these studies, promotes differentiation [10]. The idea that p63 undergoes changes in alternative splicing from p63α and -β (naive hMSC) to p63γ (differentiating hMSC) was further evaluated by transitioning hMSC from 21% to 3% O_2_ and then assessing the mRNA and protein expression of p63. Transitioning hMSC from 21% O_2_ (normoxia) to 3% O_2_ (low oxygen) led to a significant (p<0.05) decrease in the mRNA expression of *TAp63* after 24 and 48 hours (Fig 8A). A similar significant (p<0.05) decrease was seen for the mRNA expression of *p63γ*, but not for *p63α*,*β*, suggesting that low oxygen specifically decreases the mRNA expression of *TAp63γ* (Fig 8A). Following up on these results, hMSC were transitioned to 3% O_2_ for 1-week and then passaged once prior to assessing the protein levels of p63. This protocol decreased the protein expression level of TAp63γ (Fig 8B). As a proof-of-principal positive control to assess the ability of low oxygen to prevent differentiation and maintain a naive cellular state, the stem cell marker and transcription factor Oct4a, which is positively regulated by low oxygen (1–3%), was assessed [16,17]. As expected, the protein level of Oct4 increased due to low oxygen tension in the hMSC cultures (Fig 8B).

## Discussion

The results presented here demonstrate that hMSC do indeed express p63 gene products, and those gene products appears to be involved in and important for the progression of naive hMSC toward an osteoblast cell during *in vitro* osteoblastic differentiation.

### Function of endogenous p63 variants during the osteoblastic differentiation of hMSC

The p63 gene products have been shown to be involved in epithelial stem cell maintenance (TAp63) [9] and required for the development and terminal differentiation (ΔNp63) of various somatic cell lineages, including epithelial cells [9,18] and mesenchymal-derived bone cells during endochondral bone formation [1,4]. Here we show that naive, undifferentiated hMSC express predominantly TAp63α,β. During the osteoblastic differentiation of hMSC, we observed a shift in mRNA expression from TAp63α,β to TAp63γ and ΔNp63β. Under confluent conditions, known to promote differentiation of hMSC, we also observed an increase in mRNA expression of p63γ and a subsequent decrease in p63α,β. In addition, low oxygen tension (3% O_2_), known to promote the maintenance of stem cells and inhibit differentiation [10], reduced the mRNA and protein expression of TAp63γ. While overexpression of TAp63β, TAp63γ and ΔNp63β led to a loss in cell proliferation (data not shown), overexpression of ΔNp63β was shown to increase alkaline phosphatase staining—a known marker of osteoblastic cells. These studies suggest that TAp63γ and ΔNp63β are involved in the maturation and differentiation of hMSC toward the osteoblastic lineage, while TAp63α,β appear to be present only in naive, undifferentiated hMSC. Overexpression of TAp63β led to a loss in cell proliferation (data not shown), yet it was still present in naive proliferating and undifferentiated hMSC, suggesting that TAp63α is the dominant variant of p63 present in naive hMSC. Therefore, the data presented here leads to the hypothesis that the p63 variants are differentially expressed in naive hMSC (TAp63α,β) and are important during the osteoblastic differentiation of hMSC (TAp63γ and ΔNp63β). The results demonstrating that TAp63γ expression was reduced at low oxygen tension are of interest and warrant further investigation as to the ability of TAp63γ overexpression to induce differentiation even at low-oxygen tension, a situation in which hMSC are maintained in an undifferentiated state [10].

The hypothesis that TAp63γ is involved in differentiation of hMSC is further supported by a study in mouse hypertrophic chondrocyte cell lines, which demonstrated that TAp63γ increased during chondrocyte differentiation [6]. We found that TAp63γ increased during the osteoblastic differentiation of hMSC and also regulated the early pro-osteogenic genes *runx2*, *osterix*, *osteopontin* and *VDR*, which are all required for proper osteoblast differentiation. Our results strongly suggest that TAp63γ is required for the osteoblastic differentiation of hMSC. Combined, our data and previous studies suggest that TAp63γ is required for mesenchymal cell lineage commitment and differentiation in general, and not specific for either chondrocytic or osteoblastic differentiation.

There are relatively few studies that address the function(s) of the specific p63 variants during bone formation. Initial studies in p63 gene knock-out mice demonstrated that p63 was required for endochondral bone formation, but it was not clear if this was due to osteoblast dysfunction, chondrocyte dysfunction, or a combination of both [1,4]. Follow-up studies focused on the role of p63α during endochondral bone formation, in the context of chondrocyte specific overexpression of TAp63α [5] and ΔNp63α [6]. The results showed that TAp63α overexpression in chondrocytes increased ossification and osteoblast mediated mineralization in late stages of embryonic skeletal formation, tentatively due to increased matrix and collagen secretion by hypertrophic chondrocytes [5]. This suggests that TAp63α is involved in the process of endochondral bone formation through the regulation of chondrocytes, but it is not clear if there is a function in osteoblasts. Our data suggests that TAp63α is present in naive undifferentiated hMSC and decreases as they mature toward the osteoblast lineage. From these data and the literature, we hypothesize that maintenance or overexpression of TAp63α expression in naive hMSC could either (1) prevent differentiation or (2) drive the cells toward a chondrocytic phenotype.

### Function of exogenously overexpressed p63 variants during hMSC osteoblastic differentiation

It appears that during *in vitro* expansion and osteoblastic differentiation, hMSC only express four of the six p63 variants: TAp63α,β (naive hMSC, expansion), TAp63γ and ΔNp63β (osteoblastic differentiation). It is unclear from our studies if the other variants are expressed during later stages of differentiation or within the *in vivo* bone niche environment. Short-term overexpression of ΔNp63β increased alkaline phosphatase staining, supporting the observed increase in endogenous ΔNp63β during osteoblastic differentiation. Yet we also observed that short-term overexpression of ΔNp63γ, and to a lesser extent TAp63β, also increased alkaline phosphatase staining. Endogenous TAp63β expression was only present in non-confluent naive hMSC, and not during differentiation. It is possible that the beta carboxy-terminal domain of p63 (i.e., p63β) is important for the observed increase in alkaline phosphatase, which is an early osteoblastic marker. The initial increase in alkaline phosphatase during osteoblastic differentiation may be due to the basal expression of TAp63α,β in naive hMSC, and specifically a function of TAp63β. Following this line of thought, and in consideration of the fact that overexpression of TAp63β led to a loss in cell proliferation (data not shown), suggests that TAp63β is involved in the early transition of naive hMSC into alkaline phosphatase expressing pre-osteoblast like cells. Whereas, long-term overexpression of TAp63α showed no significant change in *VDR*, *osteocalcin*, or *osterix* expression, all genes involved in osteoblastic differentiation, suggesting that TAp63α is not involved in the differentiation process. In addition TAp63α did not inhibit cell proliferation (data not shown), again supporting the notion that TAp63α,β are both present in naive hMSC, and that TAp63α is not involved in differentiation while TAp63β appears to be involved in the early stages.

In a transgenic mouse model (Brdm2), which have an aberrant hypomorphic allele of p63 and only express p63γ, it was demonstrated that the alpha/beta caboxy-terminal domains were required for proper skin and limb development, whereas p63γ was not [19,20]. We hypothesize that a TAp63α,β knockout in hMSC would have dysfunctional self-renewal and maintenance of hMSC *in vitro*, which in an *in vivo* model, would result in decreased mesenchymal stem cell potency and an overall decrease in bone development and possibly limb formation, similar to Brm2 mice. Our data support the notion that TAp63γ and ΔNp63β increase during differentiation and are required for terminal osteoblastic differentiation, which is in contrast to the data seen in mice [19]. If our hypothesis is correct, the decreased limb formation in Brdm2 mice was due to mesenchymal stem cell dysfunction (lack of TAp63α,β), which would negate the role of p63γ during later stages of differentiation, since the MSC would not differentiate in the first place, hence it would appear as if p63γ had no function. Therefore, our data is in agreement with the data suggesting that alpha/beta carboxy-terminal domains are required for limb / bone development [19].

ΔNp63α and ΔNp63γ are two p63 variants which we did not detect in hMSC during expansion, or during osteoblastic differentiation. ΔNp63α is known to be required for the development of epithelial tissues [21], maintenance of epithelial stem cells [22], and important for the early stages of epithelial and keratinocyte differentiation [23,24], whereas ΔNp63γ expression is required for smooth muscle cell survival during bladder development [25]. These studies help explain why we did not detect ΔNp63α and ΔNp63γ in hMSC. Although neither ΔNp63α nor ΔNp63γ inhibited cell proliferation (data not shown), analysis of long-term overexpression studies showed a decrease in the expression of *VDR*, but with a significant increase in the expression of *osteocalcin* and *osterix*. The transcription factor osterix is required for osteoblastic differentiation of MSC and is known to up-regulate the expression of VDR in osteoblasts derived from wild type and osterix-null mice [26]. In contrast to studies reported with mice, our studies in human MSC show that ΔNp63α and ΔNp63γ increased *osterix* and decreased *VDR*. Another report, describing overexpressing p63γ in H1299 and Hela cells, and TAp63γ in an osteosarcoma cell line (SaoS2), also demonstrated a p63γ-induced increase in VDR [27]. ChIP analysis in hMSC has also demonstrated that p63 binds the VDR promoter [9]. Therefore, it appears that ΔNp63α and ΔNp63γ do indeed regulate pro-osteogenic genes and supports the idea that they have a function during the osteoblastic differentiation of hMSC.

We observed that TAp63γ expression significantly increased during osteoblastic differentiation, while ΔNp63γ was never detected. Long-term overexpression of TAp63γ stopped cell proliferation (data not shown), preventing us from determining the effect of long-term TAp63γ overexpression on VDR status. Upon short-term (7-day) knockdown of p63γ, we did observe a significant decrease in *VDR*, supporting the previously cited works that TAp63γ positively regulates the expression of VDR. From these data and the literature we speculate that TAp63γ may up-regulate or maintain, while ΔNp63γ potentially decreases the expression of VDR.

### Vitamin D_3_ metabolites differentially regulate p63 variant expression

The actions of vitamin D_3_ on bone development, health and repair are well characterized and thought to be due to a regulatory relationship between two bioactive metabolites 24R,25(OH)_2_D_3_ and 1α,25(OH)_2_D_3_. We observed that the expression of the p63 gene products are differentially regulated by the vitamin D_3_ metabolites. Specifically 1α,25(OH)_2_D_3_ increased the mRNA expression of *ΔNp63* and decreased *TAp63*, while 24R,25(OH)_2_D_3_ increased the *p63γ* splice variant mRNA. From our discussion above and the data presented, we hypothesize that TAp63γ and ΔNp63β are both required for the osteoblastic differentiation of hMSC. The 1α,25(OH)_2_D_3_ stimulated increase in *ΔNp63* mRNA suggests that 1α,25(OH)_2_D_3_ mediates its pro-osteogenic effects in part through the up-regulation of p63. Hence, the fact that *ΔNp63β* overexpression stopped cell proliferation (data not shown), while it increased alkaline phosphatase expression (both major characteristic of 1α,25(OH)_2_D_3_ / VDR activity), points toward a 1α,25(OH)_2_D_3_ - ΔNp63β mechanism.

These novel findings demonstrate for the first time that p63 variants are differentially expressed in naive hMSC (TAp63α,β), are important during the osteoblastic differentiation of hMSC (TAp63γ and ΔNp63β), and are differentially regulated by the vitamin D_3_ metabolites, 1α,25(OH)_2_D_3_ and 24R,25(OH)_2_D_3_. The molecular nuances and mechanisms of bone cell development which have been presented here will hopefully improve our understanding of bone development, complications in bone repair (mal- and non-union fracture), osteoporosis and possibly lead to new modalities of treatment.

## Materials and Methods

### Bone marrow derived mesenchymal stem cell isolation and cell culture

Primary hMSC were isolated and cultured as previously described [11], from postmortem thoracolumbar (T1-L5) vertebral bodies of donors of various ages (7–30 years old) immediately after death from traumatic injury. The results presented here are not for a single individual / donor (cell line), in that for most figures and experiments both the hMSC from a 7 (#849) and 22 (#7081) yr-old male were used. Also, two other cell lines (3 yo (#519) and 10 yo (#769)) were used in preliminary studies related to those shown here. There were no significant differences seen between the donor cells with respect to osteoblastic differentiation and/or treatment with the various vitamin D_3_ metabolites. Written informed consent from the next of kin was obtained for the use of these samples in research through collaboration with the Diabetes Research Institute, University of Miami School of Medicine (http://www.diabetesresearch.org/), which have been used in previous studies [8,10,11,14,28]. Guidelines were followed as outlined by the Committee on the Use of Human Subjects in Research at the University of Miami, and this study was reviewed and approved as exempt by Miami VA Healthcare System, Human Studies Subcommittee (IRB) (Protocol#: 1650.01). Cells were grown in Expansion media, DMEM-low glucose media containing 5% fetal bovine serum (FBS, Hyclone, Waltham, MA, Lot#30039), 20 mM ascorbic acid (Fluka/Sigma, St. Louis, MO, #49752), an essential fatty acid solution [29], and antibiotics (100 U/mL penicillin, 0.1 mg/mL streptomycin) (Gibco, Carlsbad, CA, #15140) on 10 ng/ml fibronectin (Sigma, #F2518) coated flasks (Nunclon, Rochester, NY) in 21% O_2_, 5% CO_2_, and 92% N_2_. Media was changed every 3 days, and cells were passaged upon reaching 80% confluence. For expansion hMSC were seeded at 500–1,000 cells/cm^2^.

### Osteoblastic differentiation

hMSC were initially seeded at 10,000 cells/cm^2^ overnight in expansion media [11]. Osteogenic media (α-MEM, 10% FBS, 100 U/ml-1mg/ml pen-strep, 200 μM ascorbate, 10mM β-glycero-phosphate) was used to promote osteoblastic differentiation. All flasks and wells were coated with 10 ng/ml fibronectin. Fibronectin was used consistently during expansion and osteoblastic differentiation of hMSC for two main reasons: (1) it helps maintain cell-adhesion to the plates and (2) it promotes long-term mineralization of hMSC *in vitro* [30]. Osteogenic media was added on Day 0 and changed every 3 days. The inclusion of dexamethasone (1 or 10 nM), only used in some experiments, is noted per experimental figure or result.

### Vitamin D_3_ metabolite treatment

25-hydroxyvitamin D_3_ (25(OH)D_3_) (Sigma; catalog #H4014), 24R,25-dihydroxyvitamin D_3_ (24R,25(OH)_2_D_3_) (Sigma; catalog #17943) and 1α,25-dihydroxyvitamin D_3_ (1α,25(OH)_2_D_3_) (Calbiochem; #679101) were reconstituted in ethanol (100 μM) and then stored in dark Eppendorf tubes at -80°C until added to culture media. 24R,25(OH)_2_D_3_ and 1α,25(OH)_2_D_3_ were used at 10 nM final concentration, while 25(OH)D_3_ was used at 20 nM final concentration, unless otherwise indicated. All vitamin D_3_ metabolite treatments started on Day 0 followed by media changes every 3 days for all experiments.

### Transient and stable p63 overexpression and vectors

pcDNA3.0 vectors containing each of the six p63 variants (TAp63α, TAp63β, TAp63γ, ΔNp63α, ΔNp63β, ΔNp63γ) were a gift from C. Caron de Fromentel (Lyon, France) [31]. The pcDNA3.0 vector contains neomycin/kanamycin resistant gene used for selection, and the CMV promoter and enhancer upstream of the p63 cDNA sequences. Electroporation induced transfection of hMSC with the pcDNA3.0 vector(s) was done using a Digital Bio / BTX Microporator (Digital Bio Technology, NanoEntek. Inc., Seoul, Republic of Korea). For transient overexpression of p63 variants, hMSC were allowed to recover post-transfection for 7 days in expansion media prior to osteoblastic induction with osteoblastic differentiation media. For stable overexpression of p63 variants, hMSC containing the pcDNA3.0-p63 vector were selected for with Geneticin (G418) over a 3 week period, followed by expansion and clonal selection over 2 months.

### siRNA targeted knockdown of total p63 or p63γ variant

Electroporation (Digital Bio/BTX Microporator, Holliston, MA, #MPK-1096) was used to transfect the primary hMSC with siRNA. The electroporation program consisted of 1400 V pulse voltage, 10 ms pulse width, for three pulses. SiRNA (50 & 100μM) was used for the transfection of 100,000–500,000 cells suspended in a total volume of 10μl. The siRNA targeted to knockdown (1) all p63 variants was siPANp63 (ON-TARGETplus SMARTpool Human TP73L, Dharmacon, #L-003330-00) or (2) targeted to p63γ, sip63γ: 5’-UGGAGCCCCGGAGAGUU-3’ (Dharmacon). Scrambled control siRNAs (siC) was used as a control (Dharmacon). The sip63γ siRNA targeted the unique carboxyl-terminus of p63γ knocking down both TAp63γ and ΔNp63γ. After transfection, hMSC were reseeded at 3,000 cells/cm^2^, and then allowed to attach for 3-days. Osteogenic differentiation media was added on day-3 for 4 additional days; therefore, cells were collected 7 days post siRNA transfection.

### Low oxygen culture conditions

Low oxygen tension (1–3% O_2_) has been shown to inhibit differentiation while maintaining hMSC in a naive state, while higher oxygen (normoxia, 21% O_2_), which we use in these studies, promotes differentiation [10]. hMSC were seeded at 1,000 cells/cm^2^ and grown under expansion conditions with a normoxic atmosphere (21% O_2_), and then either switched to low oxygen (3% O_2_) for 24 or 48 hours for mRNA analysis or grown at 3% O_2_ for 1-week and passaged once for protein analysis.

### RNA sample preparation and cDNA synthesis

RNA was isolated using the RNAqueous-4PCR kit (Ambion, Austin, TX, #AM1914) according to manufacturer’s directions. RNA was quantified on the Nanodrop ND-1000 Spectrophotometer (Nanodrop, Wilmington, DE), followed by reverse transcription of 2 μg RNA to cDNA using the High Capacity cDNA Reverse Transcription Kit (Applied Biosystems, Foster City, CA, #4368814), all as previously described [29].

### Real-time quantitative PCR (RT-qPCR)

Quantitative real-time PCR (RT-qPCR) was done using 10 μl of 1:20 diluted cDNA on the Mx3005P Multiplex Quantitative PCR System (Stratagene, Miami, FL, #401513) using RT-qPCR SYBR GREEN Reagents (Brilliant II SYBR Green QPCR Master Mix, Agilent Technologies, Santa Clara, CA) with ROX reference dye. A 2 μM stock solution containing both forward and reverse primer pairs was used for RT-qPCR analyses. RT-qPCR cycling conditions: 95°C for 10 min, 40 cycles at 95°C for 30 sec, 58°C for 30 sec, 72°C for 15 sec. RT-qPCR data were analyzed using the ΔΔCP method [32] and normalized against one negative control and two reference genes, eukaryotic translational elongation factor 1 alpha (*EF1α*, NM_001402) and ribosomal protein L13a (*RPL13a*, NM_01242) [8,29,33]. Results are displayed as “relative fold change” as compared to an untreated samples set to the value of “1” (dashed line in the respective figures). In the case where the “relative fold difference” of p63 variants was assessed within the same sample (Fig 1B), the primer pair efficiency was determined using serial dilutions of cDNA, to take into account the variability in primer pair efficiencies (see methods as previously published [34,35]). The calculated primer pair efficiencies (E) were as follows: *RPL13a*; 1.97, *EF1α*; 1.96, *PAN-TAp63*; 1.89, *PAN-ΔNp63*; 1.94, *TA-ΔNp63αβ*; 1.96, *TA-ΔNp63γ*; 1.98.

### Primer pairs used for RT-qPCR analyses

Primer pairs were from Operon (Eurofins MWG Operon, Huntsville, AL). Primer-BLAST (http://www.ncbi.nlm.nih.gov/tools/primer-blast/index.cgi?LINK_LOC=BlastHome) was used to construct primer pair sequences. Human primer pair sequences were as follows: *EF1α* (F: 5'-AGGTGATTATCCTGAACCATCC-3’, R: 5'-AAAGGTGGATAGTCTGAGAAGC-3’); *RPL13a* (F: 5’-CATAGGAAGCTGGGAGCAAG-3’, R: 5’-GCCCTCCAATCAGTCTTCTG-3’); *PAN-ΔNp63 (α*,*β*,*γ)* (F: 5'-TCCTGGAGCCAGAAGAAAGGACAGC-3', R: 5'-CCA GGTTCGTGTACTGTGGCTCACT-3'); *PAN-TAp63 (α*,*β*,*γ)* (F: 5'-TTCACGGTGTGCCACCCTACAGT-3', R: 5'-TGCTCTGGGACATGGTGGATCGG-3'); *TA-ΔNp63α*,*β* (F: 5'-ATCCTGGACCACCGGCAGCT-3', R: 5'-CCGGGTCTCACTGGAGCCCA-3'); *TA-ΔNp63γ* (F: 5'-CCGTGAGACTTATGAAATGCTGT-3', R: 5'-GCAGGCTGAAAG GAGATGTTT-3'); *VDR* (F: 5'-TTGACCGGAACGTGCCCCGGATC-3', R: 5'-CCTCATCTGTCAGAATGAACTC C-3'); *Osteocalcin* (F: 5'-CACTCCTCGCCCTATTGGC-3', R: 5'-CCCTCCTGCTTGGACACAAAG-3'); *BSP* (F: 5’- GAATGGCCTGTGCTTTCTCAA-3’, R: 5’- TCGGATGAGTCACTACTGCCC-3’); Osterix (F: 5’-CCCAGGCAACACTCCTACTC-3’, R: 5’-GGCTGGATTAAGGGGAGCAAA-3’); *Runx2* (F: 5’-TCCTATGACCAGTCTTACCCCT-3’, R: 5’-GGCTCTTCTTACTGAGAGTGGAA-3’); *Osteopontin* (F: 5’-ACGCCGACCAAGGAAAACTC-3’. R: 5’-AGTGCTGCTTTTCCTCAGAACT-3’).

### Alkaline phosphatase activity and staining

Cells were stained for alkaline phosphatase activity. Briefly, cells were fixed in 2% PFA/0.2% gluteraldehyde for 1h, and then incubated with substrate solution for 30 min at 37°C. Substrate solution contained 8 mg napthol AS-TR (Sigma) in 0.3 mL n-n'dimethylformamide (Sigma) mixed with 24 mg fast blue BB (Sigma) in 30 mL 100 mM Tris-HCL (pH 9.6). Subsequently, 10 mg MgCL_2_ was added, the pH was adjusted to 9.0, and then the entire solution was filtered (0.2 μM pore size).

### Protein preparation, western blot analysis, and quantification

Cell pellets were resuspended in NP40 lysis buffer (50 mM Tris, 1.0% NP40, 150 mM NaCl, 2 mM EGTA, 2 mM EDTA, 50 mM NaF, and 0.1 mM NaVO_4_, pH 8.0). Protease inhibitor cocktail (Sigma, #P8340) was added (2 μl/ml lysis buffer) to NP40 lysis buffer before protein extracts were collected. Protein concentration was determined with the BCA protein assay (Pierce, Rockford, IL). Blocking solution consisted of either 2–5% BSA or dried milk in Tris-buffered saline solution with 1–2.5% Tween 20. Antibodies used were: α-tubulin (Santa Cruz Biotechnology, Inc), TAp63 (BioLegend, Inc.; #618902), ΔNp63 (BioLegend, Inc.; #619002). The quantifiation of western blot bands was done using Image J (as described in [36]), and normalized against loading controls.

### Simon automated Simple Western assay, analysis, quantification

Simon SimpleWestern System (ProteinSimple) is an automated capillary-based technique which eliminates the blotting steps of normal Western Blots, allowing for more accurate, reproducible, and quantifiable assessment of protein levels in samples [37]. hMSC were lysed in NP40 lysis buffer (see above). Prior to loading into capillaries, a reducing buffer containing DTT with fluorescent internal molecular weight standards (1kDa, 12kDa, 180kDa; ProteinSimple; #CBS201STD1) was added to protein lysates and boiled for 5 minutes. 10 μl of protein lysate (0.1 μg/ul) was added to each capillary, with a loadtime of 17 seconds and separated (by molecular weight) for 45 minutes at 250V. Target proteins were immunoprobed with primary antibodies and horseradish peroxidase-conjugated anti-rabbit secondary antibodies (Protein Simple Antibody Detection Kit; Simon-01-01). Luminol/peroxide was used for chemiluminescence which was captured by a CCD camera (ProteinSimple; #041–271 and #041–272). Signal intensities were quantified (Compass Software; ProteinSimple) and analysed. α-tubulin was used as loading control for normalization of p63 and Oct4A protein levels. Primary antibodies used were α-tubulin (Cell Signaling Technology; #2125) (1:25 dilution), Oct4A (Abcam, Inc.; #ab18976) (1:25 dilution), TAp63 (BioLegend, Inc.; #618902) (1:25 dilution) and ΔNp63 (BioLegend, Inc.; #619002) (1:25 dilution).

### Statistical analysis

All datasets for each of the figures contained N ≥3 independent experiments with triplicate samples per condition per experiment, unless otherwise noted. One-way ANOVA followed by Tukey’s post-hoc analysis was used to calculate statistical significance between treatment conditions compared to untreated controls (GraphPad Software, San Diego, CA). Student’s *t*-test was used for direct comparisons, with p≤0.05 considered significant. Error bars represent standard deviation (SD).

## References

[1] Yang A, Schweitzer R, Sun D, Kaghad M, Walker N, et al p63 is essential for regenerative proliferation in limb, craniofacial and epithelial development. Nature. 1999;398: 714–718. 10227294 10.1038/19539

[2] Yang A, Kaghad M, Caput D, McKeon F. On the shoulders of giants: p63, p73 and the rise of p53. Trends Genet. 2002;18: 90–95. 11818141 10.1016/s0168-9525(02)02595-7

[3] Yang A, Kaghad M, Wang Y, Gillett E, Fleming MD, et al p63, a p53 homolog at 3q27-29, encodes multiple products with transactivating, death-inducing, and dominant-negative activities. Mol Cell. 1998;2: 305–316. 9774969 10.1016/s1097-2765(00)80275-0

[4] Mills AA, Zheng B, Wang XJ, Vogel H, Roop DR, et al p63 is a p53 homologue required for limb and epidermal morphogenesis. Nature. 1999;398: 708–713. 10227293 10.1038/19531

[5] Li F, Lu Y, Ding M, Wu G, Sinha S, et al Putative function of TAP63alpha during endochondral bone formation. Gene. 2012;495: 95–103. 10.1016/j.gene.2011.12.057 22244744 PMC3278498

[6] Lu Y, Abbassi S, Li F, Ding M, Wu G, et al Distinct function of P63 isoforms during embryonic skeletal development. Gene. 2013;519: 251–259. 10.1016/j.gene.2013.02.021 23481305 PMC3628837

[7] Gu J, Lu Y, Qiao L, Ran D, Li N, et al Mouse p63 variants and chondrogenesis. Int J Clin Exp Pathol. 2013;6: 2872–2879. 24294373 PMC3843267

[8] Curtis KM, Aenlle KK, Roos BA, Howard GA. 24R,25-Dihydroxyvitamin D3 Promotes the Osteoblastic Differentiation of Human Mesenchymal Stem Cells. Mol Endocrinol. 2014;28: 644–658. 10.1210/me.2013-1241 24597546 PMC4004781

[9] Chen K, Aenlle KK, Curtis KM, Roos BA, Howard GA. Hepatocyte growth factor (HGF) and 1,25-dihydroxyvitamin D3 together stimulate human bone marrow-derived stem cells toward the osteogenic phenotype by HGF-induced up-regulation of VDR. Bone. 2012;51: 69–77. 10.1016/j.bone.2012.04.002 22521434

[10] D'Ippolito G, Diabira S, Howard GA, Roos BA, Schiller PC. Low oxygen tension inhibits osteogenic differentiation and enhances stemness of human MIAMI cells. Bone. 2006;39: 513–522. 16616713 10.1016/j.bone.2006.02.061

[11] D'Ippolito G, Schiller PC, Ricordi C, Roos BA, Howard GA. Age-related osteogenic potential of mesenchymal stromal stem cells from human vertebral bone marrow. J Bone Miner Res. 1999;14: 1115–1122. 10404011 10.1359/jbmr.1999.14.7.1115

[12] D'Ippolito G, Howard GA, Roos BA, Schiller PC. Sustained stromal stem cell self-renewal and osteoblastic differentiation during aging. Rejuvenation Res. 2006;9: 10–19. 16608390 10.1089/rej.2006.9.10

[13] Colter DC, Class R, DiGirolamo CM, Prockop DJ. Rapid expansion of recycling stem cells in cultures of plastic-adherent cells from human bone marrow. Proc Natl Acad Sci. 2000;97: 3213–3218. 10725391 10.1073/pnas.070034097 PMC16218

[14] Aenlle KK, Curtis KM, Roos BA, Howard GA. Hepatocyte growth factor and p38 promote osteogenic differentiation of human mesenchymal stem cells. Mol Endocrinol. 2014;28: 722–730. 10.1210/me.2013-1286 24673557 PMC4004777

[15] Weinstein RS. Glucocorticoid-induced osteoporosis and osteonecrosis. Endocrinol Metab Clin North Am. 2012;41: 595–611. 10.1016/j.ecl.2012.04.004 22877431 PMC3417039

[16] Atlasi Y, Mowla SJ, Ziaee SA, Gokhale PJ, Andrews PW. OCT4 spliced variants are differentially expressed in human pluripotent and nonpluripotent cells. Stem Cells. 2008;26: 3068–3074. 10.1634/stemcells.2008-0530 18787205

[17] Boyer LA, Lee TI, Cole MF, Johnstone SE, Levine SS, et al Core transcriptional regulatory circuitry in human embryonic stem cells. Cell. 2005;122: 947–956. 16153702 10.1016/j.cell.2005.08.020 PMC3006442

[18] Romano RA, Smalley K, Magraw C, Serna VA, Kurita T, et al DeltaNp63 knockout mice reveal its indispensable role as a master regulator of epithelial development and differentiation. Development. 2012;139: 772–782. 10.1242/dev.071191 22274697 PMC3265062

[19] Wolff S, Talos F, Palacios G, Beyer U, Dobbelstein M, et al The alpha/beta carboxy-terminal domains of p63 are required for skin and limb development. New insights from the Brdm2 mouse which is not a complete p63 knockout but expresses p63 gamma-like proteins. Cell Death Differ. 2009;16: 1108–1117. 10.1038/cdd.2009.25 19300453 PMC2778344

[20] Talos F, Wolff S, Beyer U, Dobbelstein M, Moll UM. Brdm2—an aberrant hypomorphic p63 allele. Cell Death Differ. 2010;17: 184–186. 20161489 10.1038/cdd.2009.158 PMC2794917

[21] Kim S, Choi IF, Quante JR, Zhang L, Roop DR, et al p63 directly induces expression of Alox12, a regulator of epidermal barrier formation. Exp Dermatol. 2009;18: 1016–1021. 10.1111/j.1600-0625.2009.00894.x 19555433 PMC2857403

[22] Sareen D, Saghizadeh M, Ornelas L, Winkler MA, Narwani K, et al Differentiation of human limbal-derived induced pluripotent stem cells into limbal-like epithelium. Stem Cells Transl Med. 2014;3: 1002–1012. 10.5966/sctm.2014-0076 25069777 PMC4149305

[23] Ogawa E, Okuyama R, Egawa T, Nagoshi H, Obinata M, et al p63/p51-induced onset of keratinocyte differentiation via the c-Jun N-terminal kinase pathway is counteracted by keratinocyte growth factor. J Biol Chem. 2008;283: 34241–34249. 10.1074/jbc.M804101200 18849344 PMC2662236

[24] Warner SM, Hackett TL, Shaheen F, Hallstrand TS, Kicic A, et al Transcription factor p63 regulates key genes and wound repair in human airway epithelial basal cells. Am J Respir Cell Mol Biol. 2013;49: 978–988. 10.1165/rcmb.2012-0447OC 23837456 PMC5459548

[25] Cheng W, Jacobs WB, Zhang JJ, Moro A, Park JH, et al DeltaNp63 plays an anti-apoptotic role in ventral bladder development. Development. 2006;133: 4783–4792. 17079275 10.1242/dev.02621

[26] Zhang C, Tang W, Li Y, Yang F, Dowd DR, et al Osteoblast-specific transcription factor Osterix increases vitamin D receptor gene expression in osteoblasts. PLoS One. 2011;6(10): e26504 10.1371/journal.pone.0026504 22028889 PMC3196580

[27] Kommagani R, Caserta TM, Kadakia MP. Identification of vitamin D receptor as a target of p63. Oncogene. 2006;25: 3745–3751. 16462763 10.1038/sj.onc.1209412

[28] Delcroix GJ, Curtis KM, Schiller PC, Montero-Menei CN. EGF and bFGF pre-treatment enhances neural specification and the response to neuronal commitment of MIAMI cells. Differentiation. 2010;80: 213–227. 10.1016/j.diff.2010.07.001 20813449

[29] Curtis KM, Gomez LA, Rios C, Garbayo E, Raval AP, et al EF1alpha and RPL13a represent normalization genes suitable for RT-qPCR analysis of bone marrow derived mesenchymal stem cells. BMC Mol Biol. 2010;11: 61 10.1186/1471-2199-11-61 20716364 PMC2931506

[30] Linsley C, Wu B, Tawil B. The effect of fibrinogen, collagen type I, and fibronectin on mesenchymal stem cell growth and differentiation into osteoblasts. Tissue Eng Part A. 2013;19: 1416–1423. 10.1089/ten.TEA.2012.0523 23360404

[31] Petitjean A, Ruptier C, Tribollet V, Hautefeuille A, Chardon F, et al Properties of the six isoforms of p63: p53-like regulation in response to genotoxic stress and cross talk with DeltaNp73. Carcinogenesis. 2008;29: 273–281. 18048390 10.1093/carcin/bgm258

[32] Pfaffl MW. A new mathematical model for relative quantification in real-time RT-PCR. Nucleic Acids Res. 2001;29: e45 11328886 10.1093/nar/29.9.e45 PMC55695

[33] Studer D, Lischer S, Jochum W, Ehrbar M, Zenobi-Wong M, et al Ribosomal Protein L13a as a Reference Gene for Human Bone Marrow-Derived Mesenchymal Stromal Cells During Expansion, Adipo-, Chondro-, and Osteogenesis. Tissue Eng Part C Methods. 2012;18(10): 761–71. 22533734 10.1089/ten.tec.2012.0081 PMC3460615

[34] Curtis KM, Gomez LA, Rios C, Garbayo E, Raval AP, et al EF1alpha and RPL13a represent normalization genes suitable for RT-qPCR analysis of bone marrow derived mesenchymal stem cells. BMC Mol Biol. 2010;11: 61 10.1186/1471-2199-11-61 20716364 PMC2931506

[35] Rasmussen R. Quantification on the LightCycler Heidelberg: Springer Press; 2001: Springer Berlin Heidelberg. pp. 21–34.

[36] Schneider CA, Rasband WS, Eliceiri KW. NIH Image to ImageJ: 25 years of image analysis. Nat Methods. 2012;9: 671–675. 22930834 10.1038/nmeth.2089 PMC5554542

[37] Liu SH, Sardi S, Sonom B, Zocco D, McSweeney R, Fraser AD, Halleck AE, Li H, Smejkal GB, Munevar S, Jin JG, Kawai T, Ghiran I, McGrath JP, Whitman M, Shu-Wing NG, and Kuo WP. The Application of a Novel Nanovolume Capillary Electrophoresis-Based Protein Analysis System in Personalized & Translational MedicineResearch. J Bioanal Biomed. 2013; S3: 004 10.4172/1948-593X.S3-004

[38] van Bokhoven H, Brunner HG. Splitting p63. Am J Hum Genet. 2002;71: 1–13. 12037717 10.1086/341450 PMC384966

